# 54 Cost of Dakin’s Solution vs. Mafenide Soaks in Acute Burn Care

**DOI:** 10.1093/jbcr/irac012.057

**Published:** 2022-03-23

**Authors:** Jason Hirsch, Deanna DeHoff, Kathleen Hollowed, Elizabeth Halicki, Melanie S Condeni, Ashley Hink, Deepak Ozhathil, Steven A Kahn

**Affiliations:** Medical University of South Carolina, Charleston, South Carolina; The South Carolina Burn Center at Medical University of South Carolina, Charleston, South Carolina; South Carolina Burn Center at MUSC, Charleston, South Carolina; Medical University of South Carolina, Hanahan, South Carolina; Medical University of South Carolina, Charleston, South Carolina; Medical University of South Carolina, Charleston, South Carolina; Medical University of South Carolina, Charleston, South Carolina; Medical University of South Carolina, Charleston, South Carolina

## Abstract

**Introduction:**

The cost of health care in the United States is extremely high, with burn care being no exception to this rule. A 2016 study found that burn care costs are twice as much as the cost of non-burn related inpatient admissions, necessitating the need for cost savings. As one such measure, the authors no longer routinely use mafenide solution for burn care, and now use 0.0125% Dakin’s as a default topical irrigant, due to lower cost and less cytotoxicity. The aim of this analysis is to investigate the cost savings from using Dakin’s Solution (0.125%, 0.25%, and 0.50% strengths) versus the theoretical cost of using an equivalent amount of 5% mafenide.

**Methods:**

This study was a retrospective review that characterized a single cohort of burn patients treated with Dakin’s solution in the pre and post operative setting. Graft loss was recorded and defined as >25% loss. As a default, 0.125% Dakin’s was used, and concentration was potentially escalated based on attending judgement of wound characteristics. We qualitatively compared length of stay (LOS) index to expected for length of stay index using 1.1 hospital days per %TBSA and using 2019 NBR statistics of 3 days per %TBSA for survivors. Using costs of $37.29 (0.0125%), $40.69 (0.25%), and $38.11 (0.5%) per liter of Dakin’s versus $165.05 per liter for 5% mafenide, we looked at potential savings per patient and for the entire cohort. Average cost, median cost, and total cost of both Dakin’s solution and Mafenide were calculated. Mann Whitney Test was used to compare costs of Dakin’s versus theoretical cost of mafenide.

**Results:**

The total number of cases analyzed was 39 (n=39). The median burn size was 4% TBSA (IQR:1,6) and the median LOS was 3 days (IQR:2,8) The average cost for Dakins per patient was $721.61 versus $3172.98 had mafenide been used, p< 0.001. When all of the Dakins use was amalgamated, this represents a potential cost savings of $2451.37 per patient and $95603.43 for the entire cohort. LOS index was 0.68 with the conservative measure and 0.25 using 2019 NBR data. Only 2 patients had unplanned readmissions within 30 days. None of the patients suffered graft loss.

**Conclusions:**

Use of Dakin’s solution as an alternative to mafenide results in a significant potential cost savings compared to 5% mafenide. The patients treated with Dakins in this study spent less time in the hospital than expected compared to national averages. In addition to lower strength Dakin’s dilutions being well established as less cytotoxic, this study suggests it can save money for the burn center. Future studies should directly compare the two topicals to determine if true differences in infection, healing, or length of stay that might offset or augment cost savings emerge.

